# Nrf2 activation induces mitophagy and reverses Parkin/Pink1 knock down-mediated neuronal and muscle degeneration phenotypes

**DOI:** 10.1038/s41419-021-03952-w

**Published:** 2021-07-03

**Authors:** Sentiljana Gumeni, Eleni-Dimitra Papanagnou, Maria S. Manola, Ioannis P. Trougakos

**Affiliations:** Department of Cell Biology and Biophysics, Faculty of Biology, National and Kapodistrian University of Athens, Athens, 15784 Greece

**Keywords:** Mitophagy, Mechanisms of disease, Proteasome

## Abstract

The balanced functionality of cellular proteostatic modules is central to both proteome stability and mitochondrial physiology; thus, the age-related decline of proteostasis also triggers mitochondrial dysfunction, which marks multiple degenerative disorders. Non-functional mitochondria are removed by mitophagy, including Parkin/Pink1-mediated mitophagy. A common feature of neuronal or muscle degenerative diseases, is the accumulation of damaged mitochondria due to disrupted mitophagy rates. Here, we exploit *Drosophila* as a model organism to investigate the functional role of Parkin/Pink1 in regulating mitophagy and proteostatic responses, as well as in suppressing degenerative phenotypes at the whole organism level. We found that *Parkin* or *Pink1* knock down in young flies modulated proteostatic components in a tissue-dependent manner, increased cell oxidative load, and suppressed mitophagy in neuronal and muscle tissues, causing mitochondrial aggregation and neuromuscular degeneration. Concomitant to *Parkin* or *Pink1* knock down *cncC/Nrf2* overexpression, induced the proteostasis network, suppressed oxidative stress, restored mitochondrial function, and elevated mitophagy rates in flies’ tissues; it also, largely rescued *Parkin* or *Pink1* knock down-mediated neuromuscular degenerative phenotypes. Our in vivo findings highlight the critical role of the Parkin/Pink1 pathway in mitophagy, and support the therapeutic potency of Nrf2 (a druggable pathway) activation in age-related degenerative diseases.

## Introduction

Mitochondria are very dynamic organelles as they change their shape, size, and sub-cellular localization depending on the versatile cellular demands for adequate ATP production, optimal cellular function, and survival [1]. A common feature of neurodegenerative conditions such as Parkinson’s (PD) and Alzheimer’s (AD) disease is mitochondria dysfunction [2, 3]. PD is the second most common neurodegenerative disease, characterized by loss of dopaminergic (DA) neurons, rigidity, bradykinesia, postural instability, and tremor [4].

The maintenance of cellular mitostasis directly correlates with mitochondria quality control, which is driven by mitochondrial dynamics, mitochondrial removal, and biogenesis. A terminal response to mitochondria dysfunction is their selective destruction by mitophagy [5]; a highly conserved process, where damaged mitochondria are removed by autophagosomes and delivered for degradation to lysosomes [6]. One of the main pathways involved in this process is the Parkin/PTEN-induced putative kinase 1 (Pink1)-mediated mitophagy [6]. Pink1 is activated by auto-phosphorylation and accumulates on the outer mitochondrial membrane (OMM) of dysfunctional mitochondria triggering the recruitment of the E3 ubiquitin ligase Parkin, which then ubiquitinates several proteins of OMM, amplifying a cascade signal that culminates in mitochondrial engulfment by the autophagosome [7–9]. Parkin and Pink1 are the two most commonly mutated proteins in autosomal-recessive juvenile Parkinsonism [10, 11]. Mitophagy disruption is also a hallmark of AD [12], further supporting the importance of mitochondria quality control in neurodegeneration.

Reactive oxygen species (ROS) are main by-products of mitochondria functionality [13] and elevated intracellular ROS levels can cause damage to all cellular biomolecules [14]. A master regulator of cellular redox, and also of proteome homeostasis (referred to as, proteostasis), is the nuclear factor erythroid 2 like 2 [Nrf2; a cap “n” collar (cnc)] transcription factor [15]. Key components of the proteostatic pathways are the molecular chaperones and the two main degradation machineries, namely the autophagy lysosome-(ALP) and the ubiquitin proteasome-(UPP) pathways; ALP is mostly involved in the degradation of protein aggregates and damaged organelles [8], while UPP ensures protein synthesis quality control and degradation of short-lived regulatory proteins [16]. The 26S eukaryotic proteasome comprises a 20S core particle being bound to 19S regulatory particles. The 20S proteasome consists of four stacked heptameric rings (two α-type surrounding two β-type) with the caspase‐like (C‐L), trypsin‐like (T‐L), and chymotrypsin‐like (CT‐L) proteasome enzymatic activities located at the β1, β2, and β5 subunits, respectively [17]. Reportedly, proteasomal degradation is particularly important in PD as it degrades insoluble protein inclusions of *α*-synuclein [18]. Proteasome and mitochondria are highly interdependent, since UPP requires excessive amounts of ATP and the ubiquitinated OMM proteins are degraded by the proteasome [1]. Notably, the declined functionality of UPP and ALP is a hallmark of aging and of several age‐related diseases, including neuromuscular degeneration [19].

Additionally, to its role in redox and proteome homeostasis, Nrf2 also affects several aspects of mitostasis, including mitochondrial biogenesis, fatty acid oxidation, respiration, ATP production, and the structural integrity of the organelle [20]. Our in vivo studies in the fly model have revealed that the Nrf2 *Drosophila* ortholog, namely cncC (hereafter referred to as cncC/Nrf2), regulates mitochondria biogenesis [21], while disruption of mitochondria dynamics triggers a cncC/Nrf2-dependent proteostatic response [22]. In *C. elegans*, Nrf2 becomes activated upon inhibition of mitophagy to promote the expression of mitochondrial biogenesis and mitophagy genes [23]; in support, the mitophagic/autophagic adapter protein sequestosome-1 (SQSTM1/p62) is a Nrf2 transcriptional target [23–25].

Herein, we exploit *Drosophila* as a model organism to investigate the functional role of Parkin/Pink1 in regulating mitophagy and proteostatic responses, as well as in suppressing degeneration-like phenotypes at the whole organism level. We found that *Parkin* (known as *park* in *Drosophila*) or *Pink1* knock down (KD) increased cell oxidative load and suppressed mitophagy causing neuronal and muscle degeneration. Concomitant to *park* or *Pink1* KD activation of cncC/Nrf2, induced the proteostasis network, suppressed oxidative stress, restored (independently to ref(2)P/p62) mitophagy and largely rescued *park* or *Pink1* KD-mediated neuromuscular degenerative phenotypes.

## Materials and methods

### Flies’ culture and stocks

Flies’ stocks were maintained at 25 °C, 60% relative humidity on a 12 h light:12 h dark cycle and were fed standard medium (unless otherwise indicated). The transgenic strains w^1118^ (#5905), UAS *Pink1* RNAi (#55886), UAS *Pink1* (#51648), UAS *park* RNAi (#38333), UAS *park* (#51651), UAS *ref(2)P* RNAi (#36111), Gal4-Mef2 (#27390), UAS Mito-GFP (#8443), tub-mito-roGFP2-Orp1 (#67673); the nervous system specific Gal4-Elav (#8765) and the dopaminergic neurons Gal4-TH (#8848) were obtained from the Bloomington Stock Center. The double transgenic line UAS *Lamp1*-GFP, Actin Gal4 was kindly donated by Dr. G. Orso (University of Padova). The UAS *cncC/Nrf2*, UAS *cncC/Nrf2* RNAi and the Tubulin GeneSwitch Gal4 (tubGSGal4) flies were a gift from Prof. D. Bohmann (University of Rochester, NY, USA); the conditional driver tubGSGal4 is ubiquitously activated upon dietary administration of RU486 (320 μΜ). Mito-QC was kindly donated by Prof. A. Whitworth (University of Cambridge, UK). Given that gonads display distinct aging rates and regulation of proteostatic mechanisms as compared to adult somatic tissues [26], in all presented experiments (unless otherwise indicated) referring to adult flies only micro-dissected somatic tissues (head and thorax; equal numbers from mated male and female flies) were analyzed.

### Exposure of flies to compounds, locomotion (climbing), and longevity assays

The transgene (Tg) expression inducer RU486 (Sigma Aldrich) was directly added to the flies’ culture medium. Doses and duration of flies’ exposure to compounds are indicated in figure legends. The locomotion (climbing) and longevity assays were done as described previously [27].

### Genomic DNA extraction and conventional PCR analyses

To verify the establishment of transgenic flies carrying more than one Tg, genomic DNA from transgenic larvae or flies’ tissues was extracted with the Genomic DNA Kit of Thermo Scientific (#K0512) and conventional PCR was performed. Primers were designed using the primer-BLAST tool (http://www.ncbi.nlm.nih.gov/tools/primer-blast/) and were the following:

*Valium20*-F: ACCAGCAACCAAGTAAATCAAC, *Valium20*-R: TAATCGTGTGTGATGCCTACC; *cncC/Nrf2*-F: TGGAATTGGGCACCCATGGCG, *cncC/Nrf2*-R: AGTTTGAGTACGTCGTTCAACA.

### RNA extraction, cDNA synthesis, and quantitative Real Time PCR (Q‐PCR) analyses

Total RNA extraction from *Drosophila* tissues, cDNA preparation and Real-time Q-PCR were performed as described before [27]. Used primers have been reported before [27]; for the *Pink1*/*park* genes the primers were as follows:

*Pink1-F: ACAGCTGGTCTACAACATCC, Pink1-R: ACTGTAGGATCTCCGGACTG;*

*park-F: TTCTGCCGCAATTGTCTGCAGG, park-R: GCATGCAACCGCCATCTCGCTC;*

The *RpL32/rp49* gene expression was used as a normalizer.

### Preparation of tissue protein extracts, immunoblot analyses and detection of protein carbonyl groups

Adult or larvae tissues protein extracts preparation and immunoblotting analyses were performed as described previously [22]. For the detection of protein carbonyl groups, the OxyBlot protein oxidation detection kit (Millipore, s7150) was used.

### Measurement of ROS, proteasome, and cathepsins enzymatic activities in tissue extracts

For ROS measurement dissected flies’ tissues were incubated with fluorescent dye CM-H_2_DCFDA for 30 min at 25 °C in the dark; the assay was performed as per manufacturer’s instructions.

To measure proteasome activities, heads or somatic tissues were lysed on ice, with buffers suitable for 26S proteasome isolation (0.2% Nonidet P-40, 5 mM ATP, 10% glycerol, 20 mM KCl, 1 mM EDTA, 1 mM DDT, and 20 mM Tris, pH 7.6). Following centrifugation at 19,000×*g* (4 °C), supernatants (20 μg of protein) were used to measure (after 30 min at 37 °C) the CT-L (LLVY) and C-L (LLE) enzymatic activities by recording the hydrolysis (excitation, 350 nm; emission, 440 nm) of the fluorogenic peptides N-Succinyl-Leu-Leu-Val-Tyr-AMC (AMC = 7-amino-4-methylcoumarin) (BML-P802) and Z-Leu-Leu-Glu-AMC (BML-ZW9345) (Enzo Life Sciences; Farmingdale, NY, USA), respectively.

To measure cathepsins activity, heads or somatic flies’ tissues were homogenized in extraction buffer (1 mM dithiothreitol and 1 M Tris, pH 4.0) and the lysates were cleared at 19,000 × *g* for 15 min (4 °C). Twenty micrograms of protein were then incubated in the reaction buffer (50 mM sodium acetate, 8 mM cysteine-hydrochloride, 1 mM EDTA, pH 4.0) containing the substrate Z-Phe-Arg-AMC (BML-P139) (Enzo Life Sciences) for 30 min at 37 °C and the fluorescence was recorded (excitation, 350 nm; emission, 440 nm). Assays were performed in adult flies; equal numbers of male and female flies were used.

### Tissue preparations for immunohistochemistry and confocal laser scanning microscope (CLSM) imaging

Tissues were collected and dissected in PBS, fixed in 4% formaldehyde for 15 min, washed in PBS containing 0.3% Triton X-100 and incubated with primary antibody overnight at 4 °C. Secondary antibodies, DAPI (for nuclear visualization) or Phalloidin (for actin visualization) staining were applied for 1 h at RT and samples were mounted for viewing. Following dissection tissues were also stained with 100 μΜ LysoTracker Red (as per manufacturer’s instructions). The protocol used for the detection, imaging and processing of oxidized mitochondria was as described before [22]. Samples were viewed in a Digital Eclipse Nikon C1 (Nikon, Melville, NY, USA) CLSM equipped with 40 × 1.0 NA differential interference contrast (DIC), and 60 × 1.4 NA DIC Plan Apochromat objectives; image capturing was done using the EZC1 acquisition and images were analyzed with the CLSM software (Nikon Inc.). Z-stacks with a step size of 0.4 μm were taken using identical settings. Each stack consisted of 28–30 plane images; ~10 animals per genotype were analyzed and representative images are shown. ImageJ JACoP plugin was used for estimating co-localization of lysosomes (Lamp1-GFP) and acidic vesicles stained with LysoTracker Red.

### Antibodies and probes

Primary antibodies against the *Drosophila* proteasome subunits 20S-α (sc-65755), p42A/Rpn7 (sc-65750) and p54/Rpn11 (sc-65746); the anti-Ubiquitin (Ub) (sc-8017) and the HRP-conjugated secondary antibodies were purchased from Santa Cruz. Primary antibodies against the mitochondrial proteins ATP5a/blw (ab14748) and Ndusf3 (ab14711) were purchased from Abcam. Anti-Gapdh (G9545) and anti-Flag (F3165) antibodies were obtained from Sigma Aldrich. The anti-Rabbit-IgG Rhodamine (TRITC) conjugated antibody (711-025-152) and Alexa Fluor® 488 AffiniPure Goat Anti-Rabbit IgG (111-545-003) were from Jackson ImmunoResearch. DAPI (D1306), LysoTracker^®^ Red (L7528) and Phalloidin (P3457) were from Molecular Probes^TM^-Thermo Fisher Scientific. Anti-Tyrosine Hydroxylase (anti-TH, AB152) was purchased from Merck Millipore and the Gabarap (the fly ortholog of Atg8a; hereafter referred to as Atg8a/Gabarap) antibody (#13733) was from Cell Signaling Technology, Inc.

### Mitochondria isolation and measurement of mitochondrial respiration

Mitochondria were isolated as described before [22]. The protein content of isolated mitochondria was measured by the Bradford method and mitochondrial respiration was determined using a Clark-type oxygen electrode connected to a computer-operated Oxygraph control unit (Hansatech Instruments, Norfolk, UK) as described [22]. The respiratory control ratio (RCR) was calculated as the ratio of state 3 to state 4 (ST3:ST4).

### Statistical analyses

Experiments were performed at least in triplicate (for each biological replicate, *n* ≥ 3). Assays were done after pooling isolated male/female tissues from 10 to 20 flies, unless otherwise stated. For statistical analysis, the GraphPad Prism 7.0, the MS Excel and the Statistical Package for Social Sciences (IBM SPSS; version 23.0 for Windows, NY, USA) were used. Statistical significance was evaluated using Student’s *t*-test. Data points correspond to the mean of the independent experiments and error bars denote standard deviation (SD); significance at *P* < 0.05 or *P* < 0.01 is indicated in graphs by one or two asterisks, respectively. Gene expression was plotted vs. the respective control set to 1; in all other cases (unless otherwise indicated) control values were set to 100.

For flies’ survival curves and statistical analyses, the Kaplan–Meier procedure and log-Rank (Mantel-Cox) test were used; significance was accepted at *P* < 0.05. Statistical analyses for all presented longevity experiments are reported in Supplemental Table S1.

## Results

### KD of park or Pink1 genes in young flies mobilizes proteostatic responses, increases oxidative load, causes mitochondrial aggregation in flies’ tissues, and accelerates age-related degenerative phenotypes

**Fig. 1:**
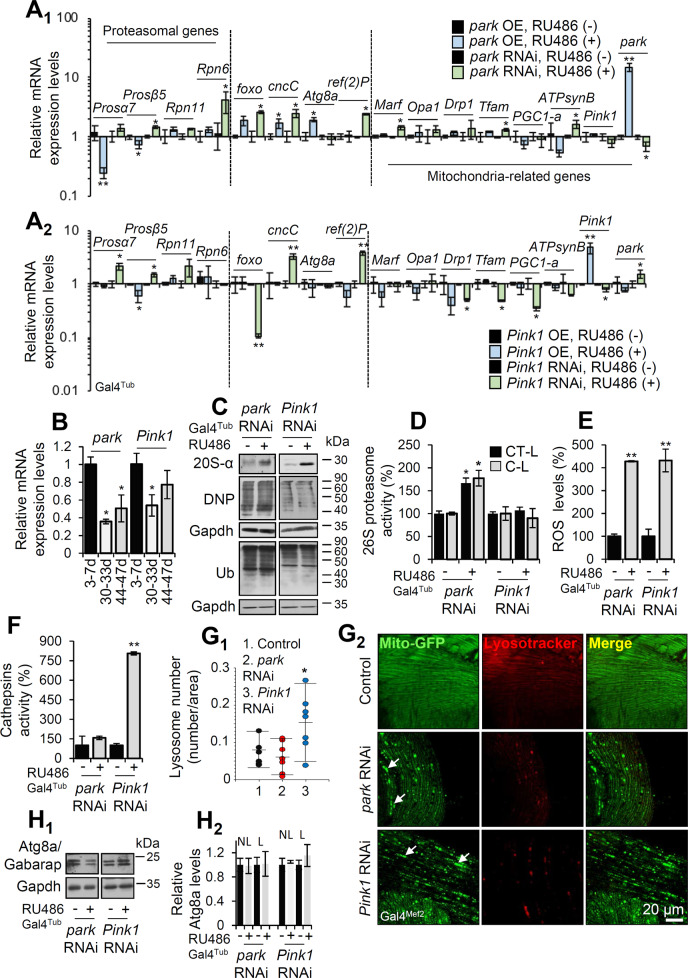
Induced KD of the mitophagy-related *park* or *Pink1* genes in flies’ tissues mobilizes proteostatic responses. **A** Relative mRNA expression of proteasomal subunits, transcription factors (*foxo, cncC/Nrf2*), autophagy related (*Atg8a, ref(*2*)P/p62*) and mitochondrial genes after inducible OE or KD of (**A**_**1**_) *park* or (**A**_**2**_) *Pink1* genes. **B** Relative mRNA expression levels of *park* and *Pink1* genes in middle aged (30–34 days old) and aged (44–47 days old) wild type flies vs. young (3–7 days old) flies. **C** Immunoblot analyses (*n* = 2) after probing the shown transgenic flies’ tissues protein samples with antibodies against 20S-α proteasomal subunits, total ubiquitin (Ub) and protein carbonylation (dinitrophenol, DNP). **D**, **E** Relative (%) 26S chymotrypsin-like (CT-L) and caspase‐like (C‐L) like proteasome activities (**D**) and ROS levels (**E**) of the shown transgenic lines. **F** Relative (%) cathepsins activity. **G** Lysosome quantification (**G**_**1**_) and CLSM co-visualization of mitochondria (Mito-GFP) and lysosomes (LysoTracker Red) (**G**_**2**_) in adult flies’ muscle tissues after muscle-targeted (Gal4^Mef2^) KD of *park* or *Pink1* genes. **H** Immunoblot analyses of (**H**_**1**_) somatic tissues probed with anti-Atg8a/Gabarap (*n* = 2) and (**H**_**2**_) relative protein quantification (NL non lipidated, L lipidated Atg8a/Gabarap). Flies were exposed to 320 μM RU486 in (**A**) for 7 days and in (**C**–**F**) for 25 days. Gene expression in (**A**, **B**) was plotted vs. control set to 1 (*RpL32/rp49* gene was used as reference). Gapdh probing in (**C**, **H**_**1**_) was used to demonstrate equal protein loading. Bars, ±SD; *n* = 3, *P < 0.05; **P < 0.01.

Initially we studied the functional crosstalk of mitostatic and proteostatic modules in young flies’ tissues, by modulating the expression of *park* or *Pink1* genes. We thus ubiquitously (Gal4^Tub^) overexpressed or knocked down *park* or *Pink1* in *Drosophila*. To assess the Tgs overexpression (OE)-mediated molecular responses we initially studied the genomic responses in transgenic flies’ somatic tissues. Gene expression analyses showed similar readouts mainly after *park* or *Pink1* KD; these referred to induction of proteasomal genes, as well as to upregulation of *cncC/Nrf2* and the mitophagy receptor *ref(*2*)P* (the fly ortholog of mammalian *SQSTM1/p62*; hereafter referred to as *ref(*2*)P/p62*), but not of *Atg8a* (the LC3 ortholog in *Drosophila*) (Fig. 1A). Some notable exceptions to similar genomic responses were *foxo* and mitochondrial genes, which tended to be induced in *park* KD but were downregulated in *Pink1* KD mutant flies (Fig. 1A).

We also found that *park* and *Pink1* are downregulated in middle aged (30–33 days old) and aged (>45 days old) wild type flies (Fig. 1B), and thus their induced ubiquitous or tissue specific KD in young flies mimics an age-related condition. Since deregulation of *park* and/or *Pink1* expression relates to human degenerative diseases [10], we focused our studies on *park*, *Pink1* KD-mediated functional outputs.

In support to gene expression analyses, 20S-*α* proteasomal proteins were upregulated after *park* or *Pink1* KD (Fig. 1C); yet *park* KD led to the accumulation of ubiquitinated (Ub) and oxidized [carbonylated (DNP)] proteins (Fig. 1C and Supplemental Fig. 1a). Although similar, these effects were milder in *Pink1* RNAi expressing mutant flies (Fig. 1C and Supplemental Fig. 1a). Studies in isolated mitochondria showed that neither *park* nor *Pink1* KD affected overall mitochondrial ubiquitination (not shown). Downstream to these effects, prolonged (25 days) ubiquitous (Gal4^Tub^) *park* KD upregulated proteasomal activities (Fig. 1D) and either *park* or *Pink1* KD significantly increased oxidative load in transgenic flies’ somatic tissues (Fig. 1E). Furthermore, by using the redox sensitive GFP (roGFPs) probe to study the in vivo oxidative load in the mitochondria of *park* and *Pink1* KD flies, we found that both transgenic fly lines tended to have increased oxidation load (Supplemental Fig. 1b, c). Also, *Pink1* KD enhanced cathepsins activity (Fig. 1F), upregulated lysosomes number (Fig. 1G), and induced Atg8a/Gabarap protein and its lipidated form expression levels (Fig. 1H); the ALP-related effects were marginal in *park* KD mutant flies (Fig. 1F, G).

**Fig. 2:**
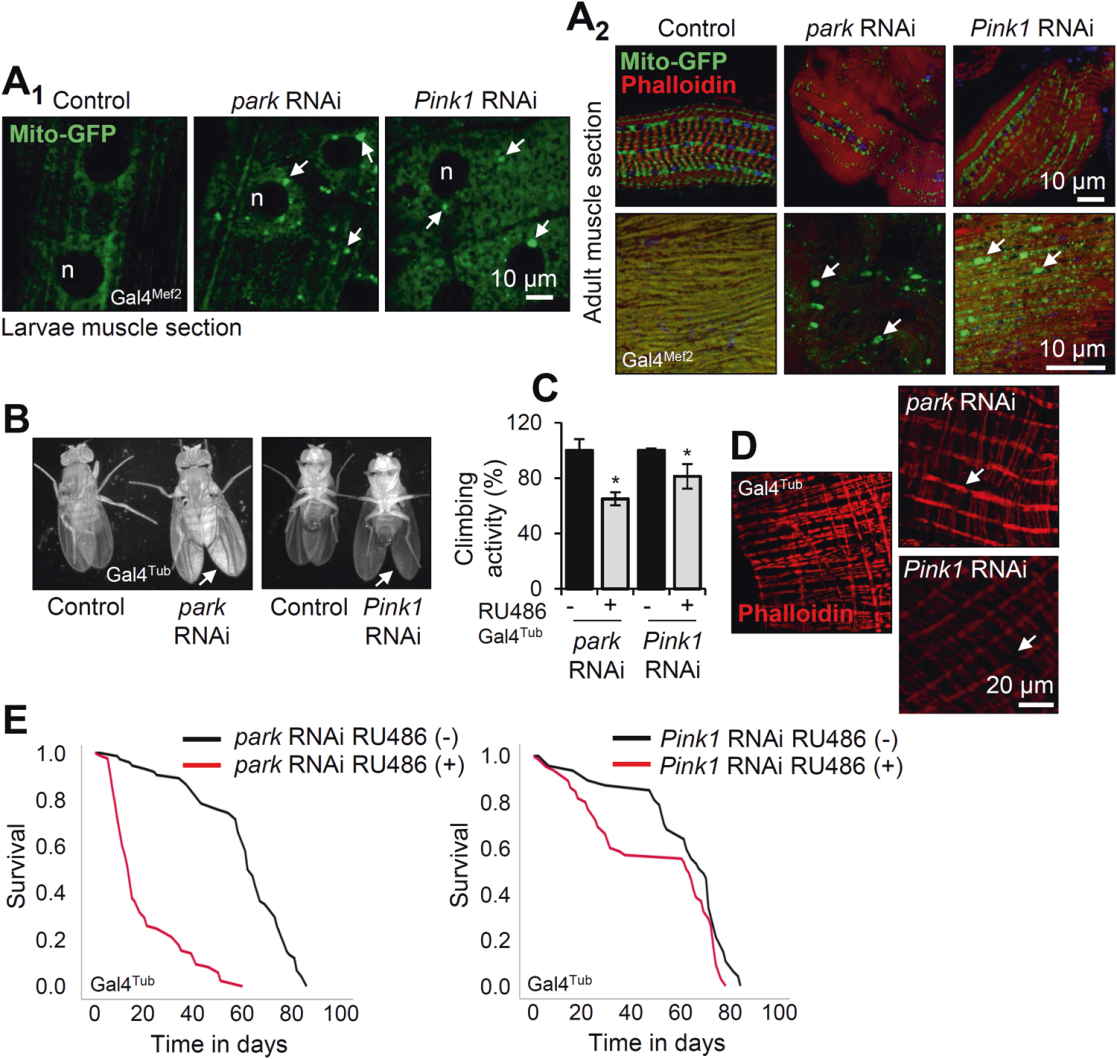
Expression of *park* or *Pink1* RNAi accelerates degenerative age-related phenotypes in transgenic flies. **A** CLSM visualization of muscle mitochondria after muscle targeted (Gal4^Mef2^) KD of *park* or *Pink1* genes in larvae (**A**_**1**_) and adult flies (**A**_**2**_) muscles; Mito-GFP was used to visualize mitochondria, phalloidin (red) for muscle actin and DAPI (blue) for cell nuclei. **B** Light stereoscope imaging of wing posture in control [RU486 (−)], *park* and *Pink1* RNAi expressing flies. **C** Relative locomotion (climbing) activity of 25 days old flies of the shown genotypes after ubiquitous (Gal4^Tub^) Tg expression. **D** Intestine muscle staining with phalloidin (red) after *park* or *Pink1* muscle targeted (Gal4^Mef2^) KD. **E** Longevity curves of the shown transgenic fly lines after ubiquitous (Gal4^Tub^) *park* or *Pink1* KD. Log-rank, Mantel-Cox test: control vs. *park* RNAi *P* < 0.0001, control vs. *Pink1* RNAi *P* < 0.01. Statistics of the longevity curves are also reported in Supplemental Table S1. Flies were exposed to 320 μM RU486 for 25 days, except otherwise indicated. Bars, ±SD; *n* = 3, *P < 0.05.

Analysis of mitochondria structure after muscle targeted (Gal4^Mef2^) Tg expression using the Mito-GFP reporter revealed the disruption of the mitochondrial network, and the increased formation of mitochondrial aggregates after *park* or *Pink1* KD (but not their OE; not shown) in both larvae (Fig. 2A_1_) and adult flies (Fig. 2A_2_) muscles; notably this genetic intervention did not significantly affect mitochondria length (Supplemental Fig. 1d). We also found the wing posture to be disrupted in young flies after muscle-targeted (not shown) or ubiquitous (Gal4^Tub^) (Fig. 2B) *park* or *Pink1* KD. This degenerative effect coincided with a significant reduction in locomotion (climbing) activity of the transgenic young flies (Fig. 2C), disruption in the organization of muscle intestine fibers (Fig. 2D) and, especially in the *park* KD mutant flies, accelerated aging (Fig. 2E and Supplemental Table S1).

Thus, ubiquitous *park* or *Pink1* KD in young flies impacts on mitostatic, proteostatic and antioxidant machineries causing their extensive readjustment, while sustained *park* or *Pink1* KD results in accumulating mitochondrial aggregates, neuromuscular degenerative phenotypes, and accelerated aging.

### Neuronal-specific or muscle-specific park, Pink1 KD disrupts proteostasis and suppresses mitophagy

**Fig. 3:**
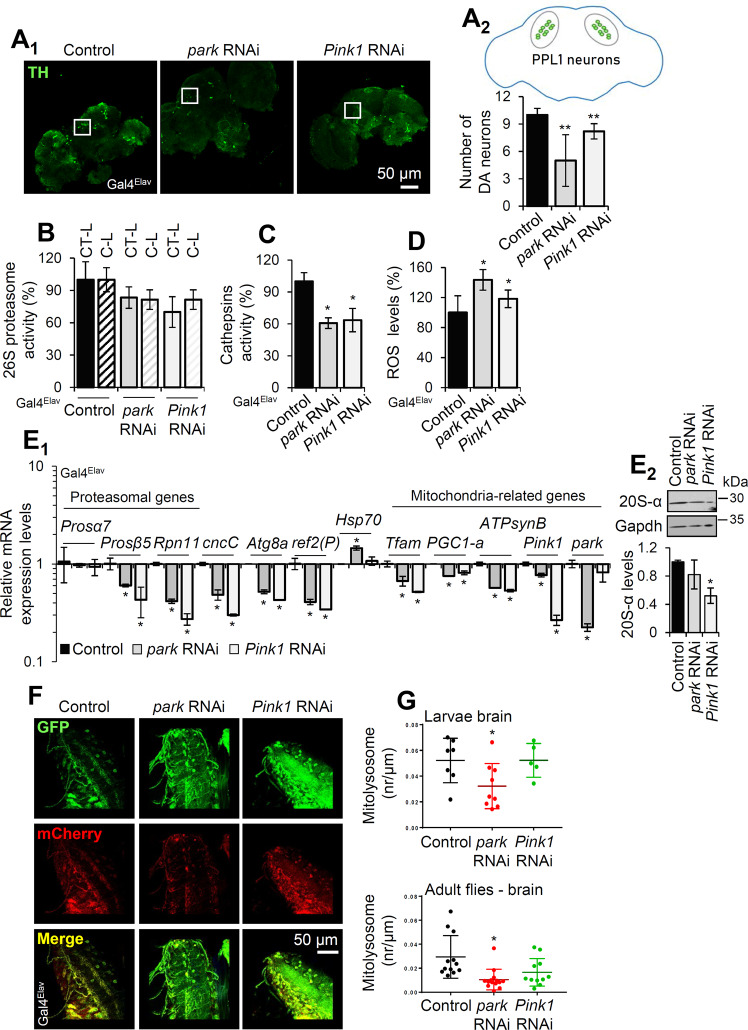
Targeted KD of *park* or *Pink1* in nervous tissues reduces brain DA neurons, downregulates proteostatic modules activities, induces oxidative stress and disrupts mitophagy. **A** CLSM viewing (projection, Z stack) of whole-mount brains from middle aged flies showing (**A**_**1**_) DA neuron clusters marked with anti-TH immunostaining, and (**A**_**2**_) quantification (vs. control) of DA PPL1 neurons in the brains of *park* or *Pink1* KD transgenic flies. **B**–**D** Relative (%) chymotrypsin-like (CT-L) and caspase‐like (C‐L) like proteasome activities (**B**), cathepsins activities (**C**), and ROS levels (**D**), in flies’ brain after KD of *park* or *Pink1* genes. **E**_**1**_ Relative mRNA expression of proteasomal subunits, *cncC/Nrf2*, autophagy-related (*Atg8a, ref(*2*)P/p62*)*, Hsp70* and mitochondria-related genes in transgenic flies of the indicated genotypes. **E**_**2**_ Immunoblot analyses and relative quantification of the shown transgenic flies’ brain samples probed with antibodies against 20S-α proteasomal subunits (*n* = 2). **F** CLSM visualization of the Mito-QC reporter GFP and mCherry signal in larvae nervous tissues, and (**G**) quantification of mitolysosomes after *park* or *Pink1* KD in larvae (upper panel) and adult flies (lower panel) brain. **A**–**G** Tgs were expressed in nervous tissues (Gal4^Elav^). Gene expression in (**E**_**1**_) was plotted vs. control set to 1; *RpL32*/*rp49* gene was used as reference. Gapdh probing in (**E**_**2**_) was used to demonstrate equal protein loading. Bars, ±SD; *n* = 3, *P < 0.05; **P < 0.01.

To assess the effects of *park* or *Pink1* genes expression silencing specifically in the brain, we expressed the *park* or *Pink1* RNAi Tgs in flies’ neuronal tissues (Gal4^Elav^). The dopaminergic (DA) network in the fly is composed of well-characterized neuron clusters, i.e., the PPM1, PPM2, PPM3, PPL1, PPL2, and VUM [28] and considering that DA neurodegeneration is a major sign of PD we immunoassayed the DA neuronal marker tyrosine hydroxylase (TH) in dissected brains after *park* or *Pink1* KD. Following prolonged (25 days) expression of the Tgs in neuronal tissues, we found a significant decrease of the DA neurons number in the PPL1 cluster (Fig. 3A), which has consistently been reported to be affected in PD fly models [29]. Further, we observed collapsed proteostasis being marked by reduced proteasomal (Fig. 3B) and lysosomal (Fig. 3C) cathepsins activities; elevated ROS levels (Fig. 3D) and a significant decrease in the expression levels of proteostatic/mitostatic genes (with the notable exception of the chaperone *Hsp70* gene induction after *park* KD; Fig. 3E1) and of 20S-*α* proteasomal proteins (Fig. 3E2) in isolated brains of *park* or *Pink1* KD transgenic flies.

To investigate the contribution of park and Pink1 proteins in neuronal mitophagy turnover rates we used the Mito-QC reporter; a tandem GFP-mCherry fusion protein of the outer mitochondrial membrane, which turns red due to selective quenching of GFP in the acidic microenvironment of the lysosome. The spectral shift results in “red-only” puncta which mark mitolysosomes [27, 30]; in these assays quantification of mitolysosomes was performed using whole brain regions after CLSM Z-stack acquisition. Mitophagy rates in control larvae were developmentally regulated (not shown) and mitophagy was more evident in live vs. fixed neuronal tissues (Supplemental Fig. 2). Our studies with the Mito-QC reporter in control flies showed that nervous tissues express high basal rates of mitophagy (Fig. 3F) and that *park* KD suppressed mitophagy, as was evident by the significant reduction (vs. controls) of mitolysosomes in both larvae and adult brains (Fig. 3F, G). *Pink1* KD in adult flies (but not in larvae) tended to also reduce mitophagy; yet the effect did not reach statistical significance (Fig. 3F, G). Consistently, neuronal specific KD of *park*, and to a lesser extent of *Pink1*, accelerated aging of transgenic flies (Supplemental Fig. 3 and Supplemental Table S1). Similarly to nervous tissues, mitophagy rates in muscles were larvae stage-dependent and more evident in live vs. fixed tissues in control flies (Supplemental Fig. 2), while as in the brain, muscle-targeted (Gal4^Mef2^) *park*, or *Pink1* KD decreased mitolysosomes number in larvae muscles, with *park* KD displaying again the stronger phenotype (Supplemental Fig. 4).

Taken together these findings highlight the toxic effects induced in young flies’ neuronal or muscle tissues (likely due to disrupted proteostasis and unbalanced mitophagy) following *park* or *Pink1* KD.

### OE of cncC/Nrf2 normalizes [independently to ref(2)P/p62)] proteostatic and mitostatic modules functionality in park or Pink1 KD transgenic flies’ tissues

Given our recent findings showing that *cncC/Nrf2* is a transcriptional regulator of mitochondrial genes [21], we then examined if *park* and/or *Pink1* genes are also regulated by *cncC/Nrf2*. We found that *cncC/Nrf2* OE induced *park* and *Pink1* (along with *ref(*2*)P/p62*) expression levels, which were suppressed after *cncC/Nrf2* KD (Supplemental Fig. 5). Thus, *cncC/Nrf2* is not only a positive regulator of *ref(*2*)P/p62*, *park,* and *Pink1* genes expression, but it also maintains their basal expression levels.

**Fig. 4:**
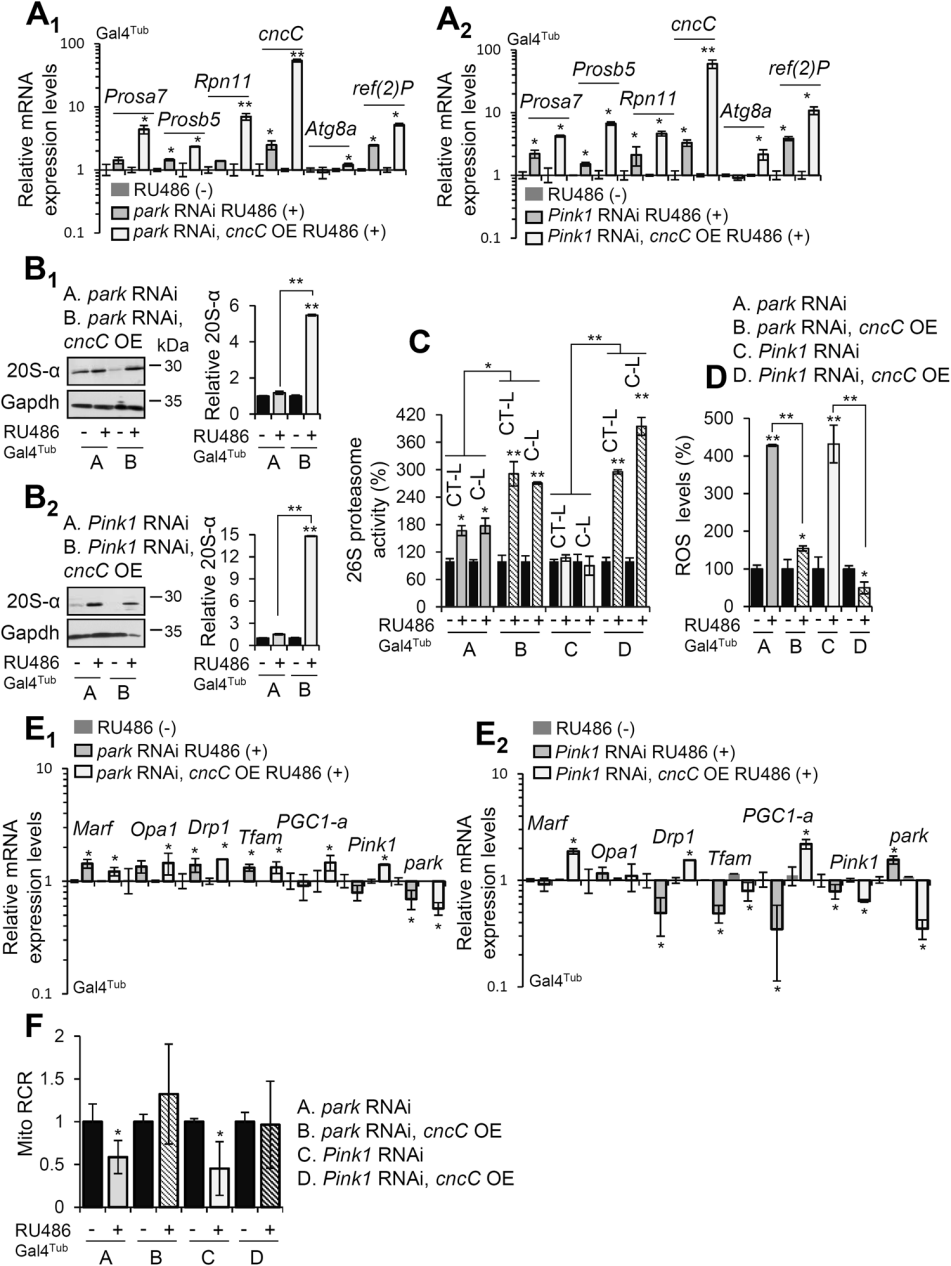
Concomitant to ubiquitous *park* or *Pink1* KD, *cncC/Nrf2* OE upregulates UPP, suppresses oxidative stress and restores normal mitochondria respiration rates in transgenic flies’ tissues. **A** Relative mRNA expression levels of proteasomal subunits (*Prosα7*, *Prosβ5*, *Rpn11*)*, cncC/Nrf2* and autophagy related (*Atg8a, ref(*2*)P/p62*) genes after *cncC/Nrf2* OE at the *park* (**A**_**1**_) or *Pink1* (**A**_**2**_) KD genetic background. **B** Immunoblot analyses and relative quantification of indicated transgenic flies’ somatic tissues probed with antibodies against 20S-α proteasomal subunits. **C**, **D** Relative (%) 26S chymotrypsin-like (CT-L) and caspase‐like (C‐L) proteasome activities (**C**), and ROS levels (**D**), following *cncC/Nrf2* OE in *park* or *Pink1* KD flies. **E** Relative mRNA expression of mitochondrial genes in shown transgenic flies. **F** Quantitative analysis of isolated mitochondria respiratory capacity at the indicated transgenic fly lines. **A**–**F** Tgs were induced ubiquitously (Gal4^Tub^); flies were exposed to 320 μM RU486. Gene expression in (**A**, **E**) was plotted vs. control set to 1 (*RpL32*/*rp49* gene was used as reference). Gapdh probing in (**B**) was used to demonstrate equal protein loading. Bars, ±SD; *n* = 3, *P < 0.05; **P < 0.01.

Considering that Nrf2 function is impaired in mitochondria-related disorders, including PD [20], and the high oxidative load in *park*, *Pink1* KD flies, we OE *cncC/Nrf2* ubiquitously (Supplemental Fig. 6a) (Gal4^Tub^) in *park* and *Pink1* RNAi expressing flies. Initially, we found that the endogenous *cncC/Nrf2* in *Drosophila*, is expressed at all developmental stages and in most *Drosophila* tissues (Supplemental Fig. 6b). OE of *cncC/Nrf2* in *park* or *Pink1* KD tissues upregulated proteasomal subunits (*Prosα*7, *Prosβ5*, and *Rpn11*), the mitophagy receptor *ref(*2*)P/p62* and *Atg8a* genes (Fig. 4A). Consistently, it also augmented 20S-α proteasomal proteins expression (Fig. 4B) and proteasomal activities (Fig. 4C); it also reduced ROS levels (Fig. 4D) and mitochondria oxidative load (Supplemental Fig. 7) as compared to transgenic flies solely expressing *park* and *Pink1* RNAi. Remarkably, OE of *cncC/Nrf2* in the *park* or *Pink1* KD background also upregulated the expression of mitochondrial genes [i.e., *Marf*, *Drp1*, and *PGC1-a* (the fly ortholog of mammalian *PPARGC1A*; peroxisome proliferator-activated receptor gamma coactivator 1-alpha)] (Fig. 4E). In addition, *cncC/Nrf2* OE upregulated the expression levels of mitochondrial complex V protein, ATP5a/blw [but not of complex I (Ndufs3)] (Supplemental Fig. 8a), and it also rescued mitochondrial respiration defects (Fig. 4F) and flies wing posture (Supplemental Fig. 8b).

**Fig. 5:**
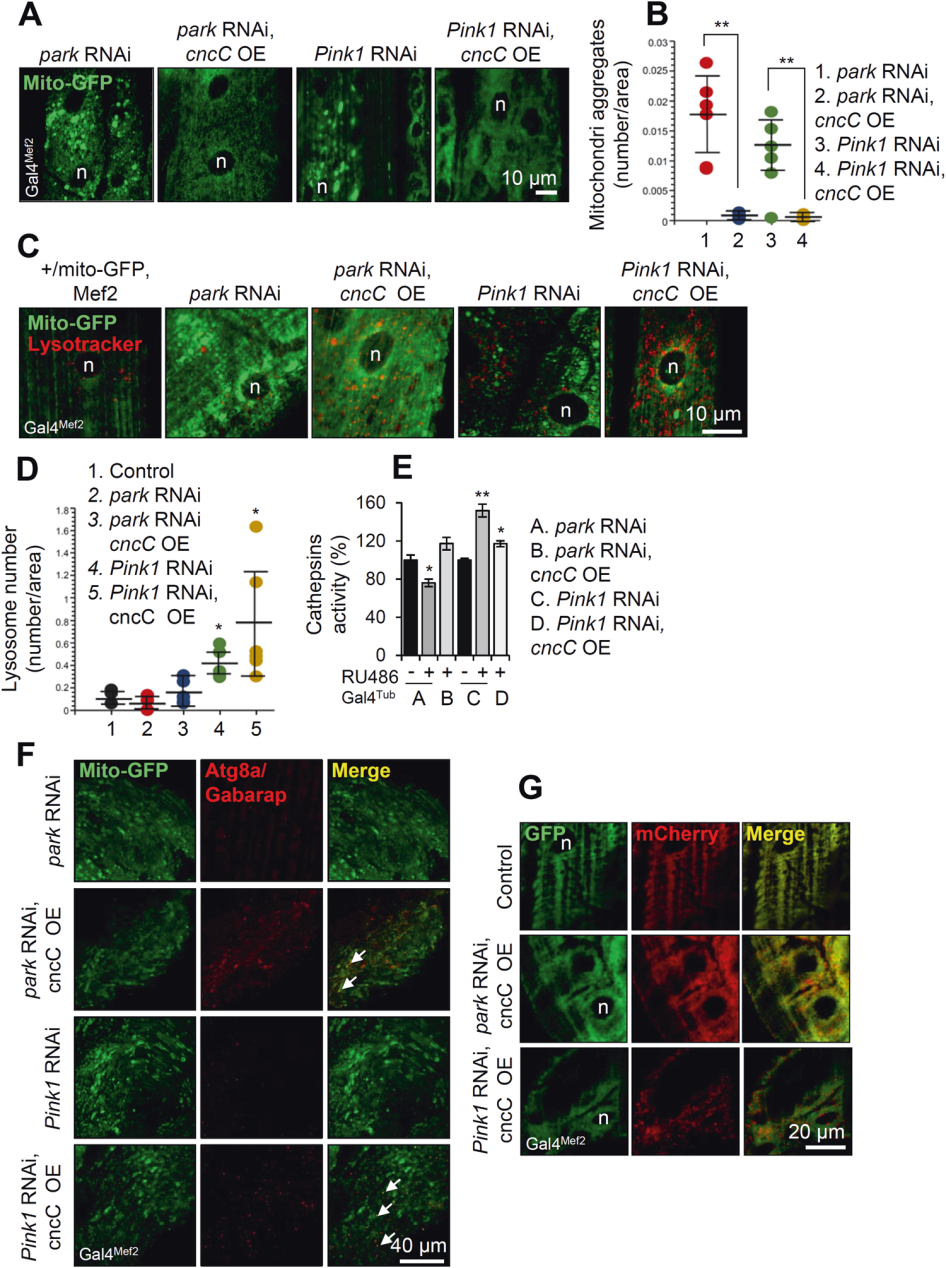
*cncC/Nrf2* OE enhances autophagy/mitophagy turnover rates in *park* or *Pink1* KD flies. **A** CLSM viewing of mitochondria (Mito-GFP), and (**B**) quantification of mitochondria aggregates after muscle-targeted (Gal4^Mef2^) expression of the shown Tgs. **C** Mitochondria (Mito-GFP), lysosomes (LysoTracker Red) co-staining and (**D**) quantification of lysosomes in the shown flies’ genotypes after muscle-targeted (Gal4^Mef2^) Tg expression. **E** Relative (%) cathepsin activity in transgenic flies after ubiquitous (Gal4^Tub^) *park* or *Pink1* KD and concomitant *cncC/Nrf2* OE. **F** CLSM viewing of mitochondria (Mito-GFP) and Atg8a/Gabarap immunostaining at the muscles (Gal4^Mef2^ driver) of shown adult transgenic flies. **G** CLSM visualization of the Mito-QC reporter GFP and mCherry signal at the indicated transgenic larvae wall muscle (Gal4^Mef2^ driver). Bars, ±SD; *n* = 3, *P < 0.05; **P < 0.01.

We thus investigated whether *cncC/Nrf2* OE could revert tissue specific disrupted mitostasis in *park* or *Pink1* KD flies. We found that muscle-targeted (Gal4^Mef2^) *cncC/Nrf2* OE eliminated Mito-GFP aggregates (Fig. 5A, B) and increased lysosomes number (Lysotracker Red staining; Fig. 5C, D) in both *park* and *Pink1* KD larvae. In support, ubiquitous *cncC/Nrf2* overexpressing flies in the *park* or *Pink1* KD background had increased cathepsins activities (Fig. 5E and Supplemental Fig. 8c), suggesting ALP stimulation. Moreover, *cncC/Nrf2* OE increased co-localization of Atg8a/Gabarap with mitochondria in larvae (not shown) and adult flies’ muscles (Gal4^Mef2^) (Fig. 5F) suggesting mitophagy stimulation. Consistently, muscle-specific expression of the Mito-QC reporter showed that *cncC/Nrf2* OE significantly enhanced (vs. controls) mitophagy in *park* and *Pink1* KD flies (Fig. 5G).

Interestingly, *ref(2)P/p62* KD in either *Park* RNAi*, cncC/Nrf2* OE or *Pink1* RNAi*, cncC/Nrf2* OE transgenic flies did not revert the *cncC/Nrf2* OE-mediated elimination of the Mito-GFP aggregates (Supplemental Fig. 9). Also, *ref(2)P/p62* KD in solely *park* or *Pink1* RNAi expressing flies tended to further increase the number of mitochondria aggregates in *park* (Supplemental Fig. 10), but not *Pink1* (not shown), KD flies indicating that *park* is more *ref(2)P/p62-*dependent for mitophagy induction. Therefore, in this biological setting cncC/Nrf2-induced mitophagy is likely independent of the autophagy/mitophagy adapter ubiquitin-binding protein ref(2)P/p62.

Thus, *cncC/Nrf2* OE activated proteostatic modules and reverted (independently to ref(2)P/p62) the formation of Mito-GFP aggregates in *park* or *Pink1* RNAi expressing flies.

### cncC/Nrf2 activation alleviates the neuromuscular degeneration phenotypes of park or Pink1 KD mutant flies

**Fig. 6:**
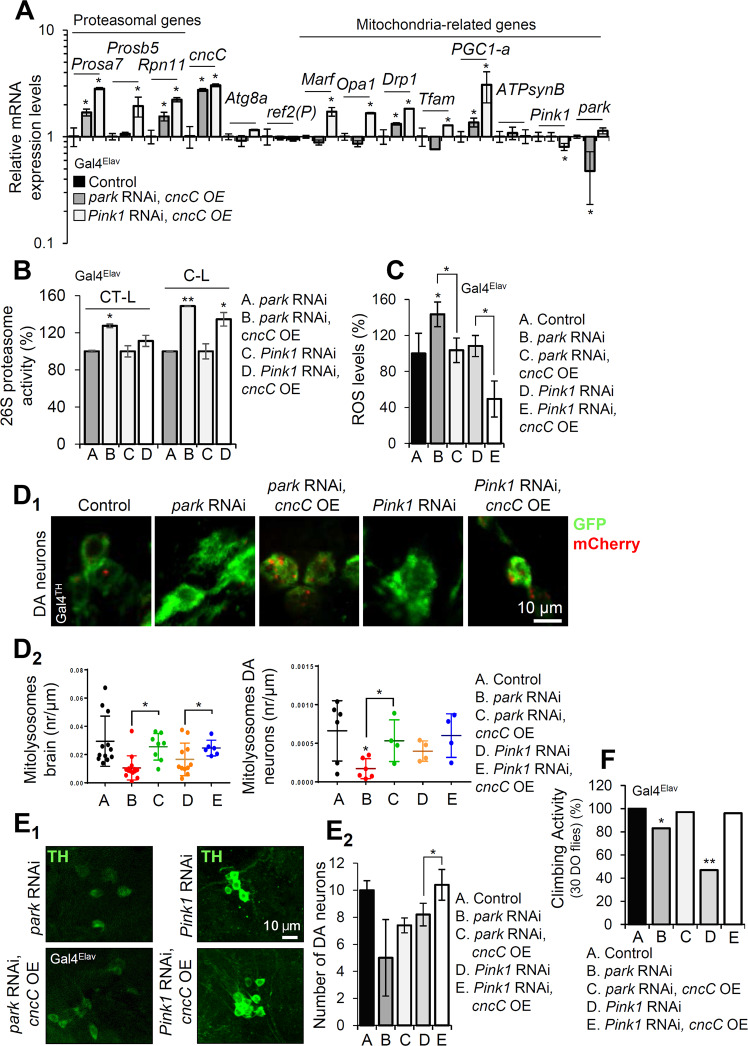
OE of *cncC/Nrf2* alleviates the neuromuscular degeneration phenotypes induced by nervous tissues-targeted *park* or *Pink1* KD. **A** Relative mRNA expression of proteasomal subunits, *cncC/Nrf2*, autophagy related (*Atg8a, ref(2)P/p62*) and mitochondrial genes in dissected brains of the indicated transgenic flies. **B**, **C** Relative (%) 26S chymotrypsin-like (CT-L) and caspase‐like (C‐L) proteasome activities (**B**), and ROS levels (**C**), (head samples) after *cncC/Nrf2* OE in *Pink1* or *park* KD flies. **D**_**1**_ CLSM visualization of the Mito-QC reporter GFP-mCherry signal in brain DA neurons (Gal4^TH^), and (**D**_**2**_) quantification of brain (Gal4^Elav^), DA (Gal4^TH^) mitolysosome number of the shown adult transgenic flies. **E**_**1**_ CLSM images of DA PPL1 neurons (middle-aged flies) following staining of dissected brains with anti-TH antibody, and (**E**_**2**_) quantification of DA PPL1 neurons at the indicated adult transgenic flies’ brain. **F** Locomotion (climbing) activity of the shown transgenic flies. In (**A**–**F**) Tgs were expressed specifically in the nervous tissues (Gal4^TH^ and/or Gal4^Elav^). Gene expression in (**A**) was plotted vs. control set to 1; *RpL32*/*rp49* gene was used as reference. Bars, ±SD; *n* = 3, *P < 0.05; **P < 0.01.

Finally, we asked whether *cncC/Nrf2* OE could also rescue the neuromuscular degeneration phenotypes manifested in *park* or *Pink1* KD flies. Gene expression analyses in isolated brains after neuronal-targeted (Gal4^Elav^) co-expression of the Tgs showed that targeted *cncC/Nrf2* OE upregulated proteasomal subunits and mitochondrial genes but not *ref(2)P/p62* and *Atg8a* (Fig. 6A; see also Fig. 3E_1_), indicating tissue-specific responses after *cncC/Nrf2* OE. Importantly, *PGC1-a* was also induced by *cncC/Nrf2* OE (Fig. 6A). Previous reports have shown that apart from a master regulator of mitochondria biogenesis, *PGC1-a* also exerts a neuroprotective role [31]. Also, *cncC/Nrf2* OE in nervous tissues increased proteasome activities (Fig. 6B), reduced oxidative load (Fig. 6C) and as found in muscles, enhanced the overall mitophagy turnover rates in *park* or *Pink1* KD flies’ brain (Fig. 6D). In support, it prevented loss of DA neurons in the brain of *park* or *Pink1* KD flies (Fig. 6E), rescued the *park*, *Pink1* KD mutant’s locomotion defects (Fig. 6F) and tended to improve, especially in the *park* KD background which showed the more severe phenotype, young flies’ health-span (Supplemental Fig. 11 and Supplemental Table S1). Yet, consistently to our recent findings showing that prolonged *cncC/Nrf2* OE in the fly can be toxic [21] the overall longevity of these transgenic flies was not (vs. controls) improved (Supplemental Fig. 11 and Supplemental Table S1).

Taken together, these data support the notion that activation of the druggable cncC/Nrf2 pathway can improve the severe effects of age-related park or Pink1 reduced functionality.

## Discussion

UPP and ALP play a critical role in regulating cellular functionality and viability and the decline of their activity is a hallmark of aging, and of multiple age-related diseases including neuromuscular degenerative disorders [16, 32]. Seemingly, there is an extensive functional wiring between antioxidant, proteostatic, and mitostatic modules [21, 22, 29, 33] and both antioxidant and proteostatic pathways curate mitochondrial functionality by triggering adaptive repair responses, mitochondrial biogenesis, and/or even removal of the damaged organelle by mitophagy; insufficiency of these responses can induce cell death [34].

We report that *park* or *Pink1* KD in young flies modulated proteostatic modules in a tissue-dependent manner; ubiquitously increased cell oxidative load (shown also after loss-off PINK1 function in SH-SY5Y human neuroblastoma cells [35]), and suppressed mitophagy in neuronal and muscle tissues causing mitochondrial aggregation and neuromuscular degeneration. Pink1 exploits the canonical mitochondrial import pathway and under physiological conditions faces proteasome degradation [36], while the E3 ubiquitin ligase Parkin is one of the numerous UPP components recruited at the OMM upon mitochondrial damage [7, 37]. The mitochondrial localization of these components is another indication of the linkage between proteostatic-mitostatic modules; a notion that is further supported by the finding that ~62% of the mitochondrial proteome might be ubiquitinated for UPP degradation [33]. Notably, *park* KD led to increased proteasomal enzymatic activities, while, Parkin-mediated mitophagy is reduced when proteasome is inhibited and proteasomes accumulate on mitochondria during Parkin-induced mitophagy [38, 39]. Thus, proteasomal activities are tightly regulated as a response to altered functionality of Parkin. On the other hand, it has been reported a deficit in proteasome activity in *Pink1* loss-of-function cells and it was suggested that this readout may relate to reduced ATP production that *Pink1* loss-of-function cells displayed [40]. Considering that both *park* and *Pink1* KD mutant flies showed similar mitochondrial oxidative phosphorylation capacity, we hypothesize that the differential effects on proteasome activities in *park*, *Pink1* KD flies may also relate to the different ubiquitination profiles that was induced in flies’ tissues after *Pink1* or *park* KD. Indeed, UPP degradation rates depend on proteome ubiquitination and the balance between ubiquitination/de-ubiquitination of the substrates [41].

Distinctly to *park* KD, downregulation of *Pink1* upregulated Atg8a/Gabarap expression and lysosomal cathepsins activity, indicating the activation of autophagic responses; in support PINK1 knockout in mouse embryonic fibroblasts resulted in autophagy upregulation [42]. Yet, in either *park* or *Pink1* KD mutant flies’ tissues there was minimal co-localization between ALP components and mitochondria aggregates, further supporting the notion that mitophagy is regulated distinctly from bulk autophagy [43]. In our study in the fly model, *Pink1* KD showed less intense phenotypes as compared to *park* KD. We hypothesize that this may relate to the fact that park functions downstream of Pink1 [28] and thus, in *Pink1* RNAi flies the remaining active protein could partially activate park. Given also the differential readouts in terms of proteostatic responses and levels of tissue oxidative load upon ubiquitous or tissue-targeted *park* or *Pink1* KD, it is likely that mitophagy displays (at least in flies) tissue-dependent regulation. However, we cannot exclude that the noted *park* or *Pink1* genes silencing effects could be partially independent of their role in mitophagy [44, 45].

Among the most challenging questions regarding mitochondrial removal is the role of Parkin/Pink1 in basal mitophagy. Similarly, to previous reports in *C. elegans* [46] we found high basal mitophagy rates in flies’ tissues, which was nonetheless marked by tissue-dependency as basal mitophagy was higher in neuronal vs. muscle tissues. Previous studies suggested lack of basal mitophagy in *Drosophila* adults or pupae muscles [30]; yet we found increased rates of basal mitophagy in larvae muscles. This discrepancy likely relates to our observation that detection of mitophagy is affected by larvae developmental stage or tissue preparation. In support, lysosomes are abundant in flies’ muscles [47] and it was suggested that their detection using probes should be made in live tissues [48]. Given the impact of tissue preparation or the developmental stage on mitophagy recording, the molecular mechanisms of this pathway, under basal or stress conditions, should be preferentially studied at in vivo models [46]. To this end, the analysis of transgenic animals expressing mitophagy reporters, such as Mito-QC, will further increase our understanding on mitophagy regulation.

Muscles were particularly sensitive to reduced *park* or *Pink1* expression as we found that KD of *Pink1* and especially of *park* reduced mitophagy events in muscles, a tissue of high metabolic demand. This phenotype explains the excessive accumulation of mitochondrial aggregates in transgenic flies’ muscles along with the muscle related degenerative phenotypes seen in young flies, i.e. disrupted wing posture and locomotion activity. Interestingly, our findings indicate that the role of *Pink1* in *Drosophila* brain is dispensable for basal mitophagy, differently from *park* which (as in muscles) seemingly plays an important role on driving mitophagy events also in neuronal tissues. In support, the role of *Pink1* in basal mitophagy is dispensable in several mouse tissues [49]. Up to date, little is known about the regulation of mitophagy in neurons and the functional involvement of the *park/Pink1* pathway in this tissue. Given the high dependence of neurons on proper mitochondria functionality, one would assume that these cells have evolved additional pathways to remove damaged mitochondria [50]. Indeed, several studies have demonstrated the existence of *park*/*Pink1*-independent mitophagy cascades revealing multiple mitochondrial proteins (FUNDC1, BNIP3, NIX, and PHB2) or even lipids (e.g., cardiolipin) that mediate mitophagy in response to different stimuli [51–53].

Given the impact of mitochondrial dysfunction on neurodegeneration disorders we sought to rescue the *Pink1* or *park* KD-mediated deleterious effects. We found that concomitant to *park* or *Pink1* KD, activation of the *cncC/Nrf2* transcription factor [20, 21], ubiquitously induced the proteostasis network, suppressed oxidative stress and restored mitochondrial function and mitophagy rates in flies’ tissues; furthermore, our data indicate that *cncC/Nrf2* is a transcriptional regulator of *park* and *Pink1* genes. Reportedly, a subset of the cytoplasmic Nrf2/Keap1 ROS-sensing complexes are tethered to OMM via the interaction of Keap1 with the mitochondrial protein PGAM5 [54], while in *C. elegans*, Nrf2 coordinates both mitochondrial removal and biogenesis upon oxidative stress [55]. Interestingly, 1,4-diphenyl-1,2,3-triazole (PMI), was found to mediate LC3 recruitment to mitochondria and to increase mitochondrial p62 co-localization in a Nrf2-dependent way [56]. Our data showed that cncC/Nrf2 can mediate mitophagy at the *park* or *Pink1* KD genetic backgrounds in a ref(2)P/p62-independent manner, suggesting that Nrf2 likely engages additional ubiquitin receptors to mediate mitophagy. Indeed, Nrf2 is a transcriptional activator in mammalian cells of the autophagy receptor NDP52 [57]; yet the identification of Nrf2-inducible receptors in *Drosophila* mitophagy should await further studies.

Finally, we observed that Nrf2 activation in the fly model largely rescued *park* or *Pink1* KD-mediated neuromuscular degenerative phenotypes. Specifically, *cncC/Nrf2-*targeted activation in neuronal tissues expressing *park* or *Pink1* RNAi, reduced oxidative load, tended to increase mitophagy and rescued loss of DA neurons in the brain. In DA neurons the park/Pink1 pathway is overactive, probably because of the high mitostatic stress that these cells face [58]; also, increased mitophagy rates due to *cncC/Nrf2* OE in surrounding to DA neurons cells, could establish a by-stander effect that reduces oxidative load and provides survival signals. Consistently, Nrf2-activating molecules such as sulforaphane or RTA-40 can restore mitochondrial membrane potential and have protective effects in neurodegeneration [59]. Thus, Nrf2 activation can represent a promising therapeutic intervention in degenerative diseases.

## Availability of Data and Materials

The datasets generated and/or analyzed during the current study are available from the corresponding author on reasonable request.

## Supplementary information

Supplemental Info
