# Supplementary material for: Nrf2 activation induces mitophagy and reverses Parkin/Pink1 knock down-mediated neuronal and muscle degeneration phenotypes
Source: Cell Death Dis. 2021 Jul 3;12(7):671. doi: 10.1038/s41419-021-03952-w (PMC8254809; doi:10.1038/s41419-021-03952-w)
Supplement: Supplementary file 1 — Supplemental Info [file 41419_2021_3952_MOESM1_ESM.pdf]

Nrf2 activation induces mitophagy and reverses Parkin/Pink1 knock down-mediated neuronal and muscle degeneration phenotypes

Sentiljana Gumeni, Eleni-Dimitra Papanagnou, Maria S. Manola, Ioannis P. Trougakos

Supplementary information

Figure Legends

Supplemental Fig. 1. Impact of *park* or *Pink1* KD on oxidative levels and mitochondria length. a Relative quantification of total ubiquitinated protein levels after *park* or *Pink1* KD (n=2). **b** CLSM visualization images of roGFP2-mito-Orp1 (405 nm and 488 nm excitation) in third instar *Drosophila* proximal muscle section. **c** 405/488 excitation ratios of roGFP2-mito-Orp1 in total protein tissues' lysates ($Gal4^{Tub}$ driver). **d** Mitochondria length (μm) of the indicated genotypes. Bars, ± *SD*; $n \geq 3$, *$P < 0.05$.

Supplemental Fig. 2. Detection of the Mito-QC reporter in fixed or live tissues of wild type *Drosophila* larvae. CLSM visualization of Mito-QC reporter GFP and mCherry signals in third instar *Drosophila* proximal nerve section ($Gal4^{Elav}$ driver) and larvae muscle ($Gal4^{Mef2}$ driver).

Supplemental Fig. 3. Targeted *park* or *Pink1* KD in flies' neuronal tissues accelerates aging. Longevity curves of *park* or *Pink1* RNAi expressing flies after targeted expression of the Tg in neuronal ($Gal4^{Elav}$) tissues. Log-rank, Mantel-Cox test: control *vs. park* RNAi, $P < 0.0001$, control *vs. Pink1* RNAi $P < 0.035$. Statistics of the longevity curves are also reported in Supplemental Table S1.

Supplemental Fig. 4. Reduction of the Mito-QC reporter signal after *park* or *Pink1* targeted KD in flies' muscle tissues. a CLSM visualization of the Mito-QC reporter GFP and mCherry signals

in third instar larvae wall muscle (Gal4[Mef2] driver). **b** Quantification of mitolysosomes of the shown in **a** transgenic larvae muscle. n, nucleus. Bars, ± *SD*; n ≥ 3, *P < 0.05.

Supplemental Fig. 5. The *ref(2)P/p62, park* and *Pink1* genes are *cncC/Nrf2* transcriptional targets in flies' tissues. a Relative mRNA expression levels of *ref(2)P/p62, park* and *Pink1* genes following targeted *cncC/Nrf2* OE or KD in neuronal tissues (Gal4[Elav]). **b** CLSM visualization of mitochondria (Mito-GFP reporter) and ref(2)P/p62 staining in control and *cncC/Nrf2* overexpressing [muscle (Gal4[Mef2]) targeted] flies. Gene expression in **a** was plotted *vs*. control set to 1 (*RpL32*/*rp49* gene was used as reference). n, nucleus. Bars, ± *SD*; n ≥ 3, *P < 0.05.

Supplemental Fig. 6. *cncC/Nrf2* expression levels at the transgenic fly line and at control (WT) flies' (different developmental stages) tissues. $\mathbf{a_1}$ Relative mRNA (*vs*. WT flies) and $\mathbf{a_2}$ protein expression levels (*vs*. the non-induced control) of the flag tagged *cncC/Nrf2* in transgenic flies (n=2) after inducible ubiquitous expression (Gal4[Tub] driver). Expression of tagged *cncC/Nrf2* is also observed at low levels in non-induced flies [RU486 (-)] due to leaky expression of the Tg in the absence of the inducer (RU486)[21]. **b** Endogenous *cncC/Nrf2* mRNA expression levels in distinct *Drosophila* developmental stages and in adult tissues. Gene expression in $\mathbf{a_1}$ was plotted *vs*. control set to 1; in both $\mathbf{a_1}$ and **b** *RpL32*/*rp49* gene was used as reference. Flies were exposed to 320 μM RU486. Bars, ± *SD*; n ≥ 3, *P < 0.05.

Supplemental Fig. 7. *cncC/Nrf2* OE reduces mitochondria oxidative load of *park* or *Pink1* KD flies. a CLSM visualization images of roGFP2-mito-Orp1 (405 nm, 488 nm excitation) in third instar *Drosophila* proximal muscle section, and **b** 405/488 excitation ratios of roGFP2-mito-Orp1 in total protein tissues' lysates (Gal4[Tub] driver) of the shown genotypes. Reference roGFP2-Mito-Orp1 micrographs and 405/488 ratio values are as in Supplemental Figs 1b, 1c. Bars, ± *SD*; n ≥ 3.

Supplemental Fig. 8. *cncC/Nrf2* OE ameliorates *park* or *Pink1* KD-related phenotypes. a Immunoblot analyses after probing the shown transgenic flies' mitochondria samples with antibodies against ATP5a/blw (complex V) and Ndusf3 (complex I) proteins (n=2). Ponceau staining was used for equal loading. $\mathbf{b_1}$ Imaging and quantification $\mathbf{b_2}$ of wing posture in control [RU486 (-)], *park* RNAi, *park* RNAi, *cncC* OE, *Pink1* RNAi, and *Pink1* RNAi, *cncC* OE expressing transgenic flies. $\mathbf{c_1}$ co-visualization of lysosomes in shown genotypes after Lamp1-

GFP expression and LysoTracker Red (labels acidic organelles) staining and **c$_2$** measurement of lysosomes (Lamp1-GFP) and acidic vesicles (stained with LysoTracker Red) co-localization. Flies were exposed to 320 µM RU486. Bars, ± *SD*; n ≥ 3, *P < 0.05, **P < 0.01.

Supplemental Fig. 9. *cncC/Nrf2*-mediated mitophagy is independent of ref(2)P/p62. a CLSM viewing of mitochondria (Mito-GFP reporter). **b** Quantification of mitochondria aggregates following muscle targeted (Gal4^{Mef2}) *ref(2)P/p62* KD in *Park* RNAi, *cncC/Nrf2* OE or *Pink1* RNAi, *cncC/Nrf2* OE transgenic flies. n, nucleus. Bars, ± *SD*; n ≥ 3, **P < 0.01.

Supplemental Fig. 10. Mitochondria aggregates after muscle-targeted simultaneous *park* and *ref(2)P/p62* KD. CLSM visualization of mitochondria (Mito-GFP reporter) in third instar *Drosophila* larvae wall muscle (Gal4^{Mef2}) of the shown genotypes. n, nucleus. Arrows indicate aggregated mitochondria.

Supplemental Fig. 11. Longevity curves (*vs*. respective controls) after *cncC/Nrf2* OE in *park* or *Pink1* KD flies. Longevity curves of the indicated transgenic fly lines after targeted expression of the Tgs in flies' nervous tissues (Gal4^{Elav}); reference curves are as in Supplemental Fig. 3. Log-rank, Mantel-Cox test: control *vs. park* RNAi P < 0.0001, control *vs. Pink1* RNAi P < 0.035, control *vs. park* RNAi, *cncC/Nrf2* OE P < 0.0001, control *vs. Pink1* RNAi, *cncC/Nrf2* OE P < 0.0001. Statistics of the longevity curves are also reported in Supplemental Table S1.

List of *Drosophila* genes

Atg8a (Autophagy-related 8a, FBgn0052672, CG32672); *ATPsynB* (ATP synthase, subunit B, FBgn0019644, CG8189); *cncC* (cap-n-collar isoform-C, FBgn0262975, CG43286); *Drp1* (Dynamin related protein 1, FBgn0026479, CG3210); *foxo* (forkhead box, sub-group O, FBgn0038197, CG3143); *Hsp70* (Heat-shock-protein-70Bb/a, FBgn0013278/FBgn0013277, CG31359/CG31449); *Marf* (Mitochondrial assembly regulatory factor, FBgn0029870, CG3869); *Opa1* (Optic atrophy 1, FBgn0261276, CG8479); *park* (parkin, FBgn0041100, CG10523); *Pink1* (PTEN-induced putative kinase 1, FBgn0029891, CG4523); *Prosα7* (Proteasome α7 subunit, FBgn0023175, CG1519); *PGC1-a/srl* (Peroxisome proliferator-activated receptor gamma coactivator 1-alpha/spargel, FBgn0037248, CG9809); *Prosβ5* (Proteasome β5 subunit,

FBgn0029134, CG12323); *ref(2)P* (refractory to sigma P, FBgn0003231, CG10360); *RpL32/Rp49* (Ribosomal protein L32, FBgn0002626, CG7939*)*; *Rpn6* (Regulatory particle non-ATPase 6, FBgn0028689, CG10149); *Rpn10* (Regulatory particle non-ATPase 10, FBgn0015283, CG7619); *Rpn11* (Regulatory particle non-ATPase 11, FBgn0028694, CG18174); *TFAM* (mitochondrial transcription factor A, FBgn0038805, CG4217).

Abbreviations

Autophagy-lysosome pathway, ALP; cap'n'collar, cnc; cap'n'collar isoform C, cncC; caspase-like activity, C-L; chymotrypsin-like activity, CT-L; dopaminergic neurons, DA; kelch-like ECH-associated protein 1, Keap1; nuclear factor erythroid 2 like 2, Nrf2; Parkinson's disease, PD; sequestosome-1, SQSTM1/p62; PTEN-induced putative kinase 1, Pink1; trypsin-like activity, T-L; Ubiquitin-proteasome pathway, UPP.

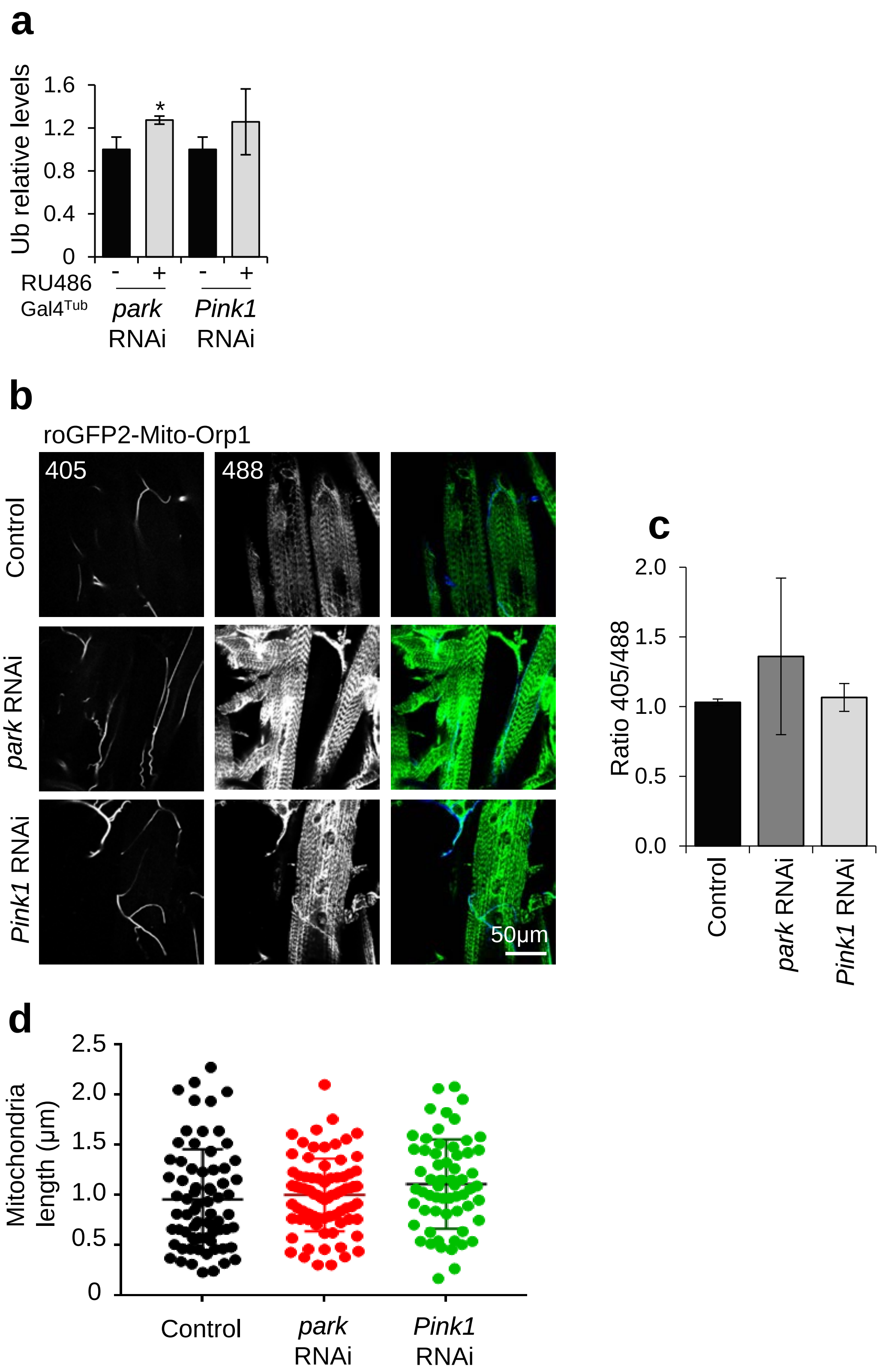

Supplemental Fig. 1

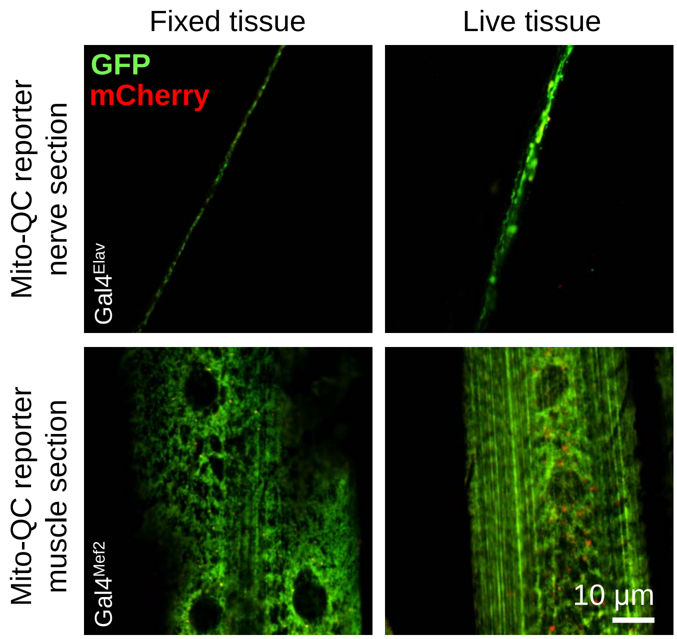

Supplemental Fig. 2

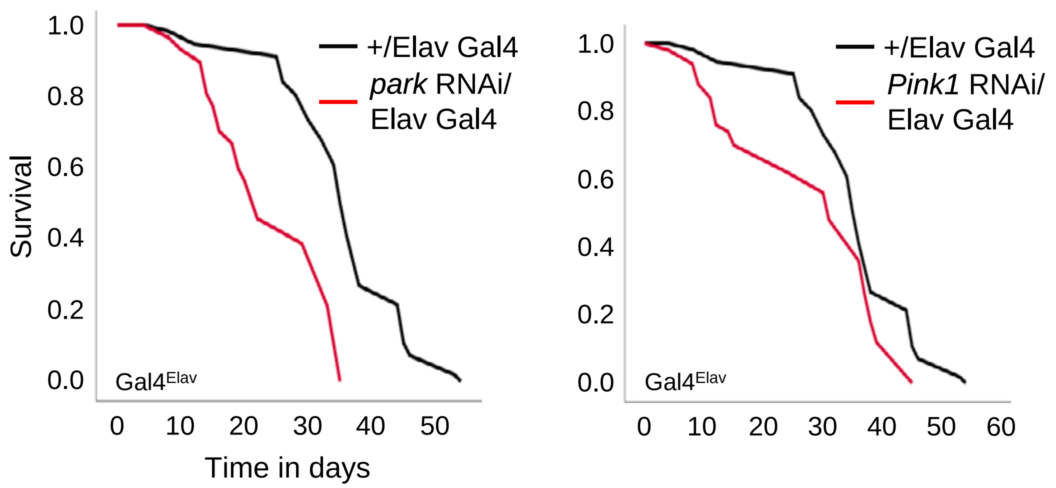

Supplemental Fig. 3

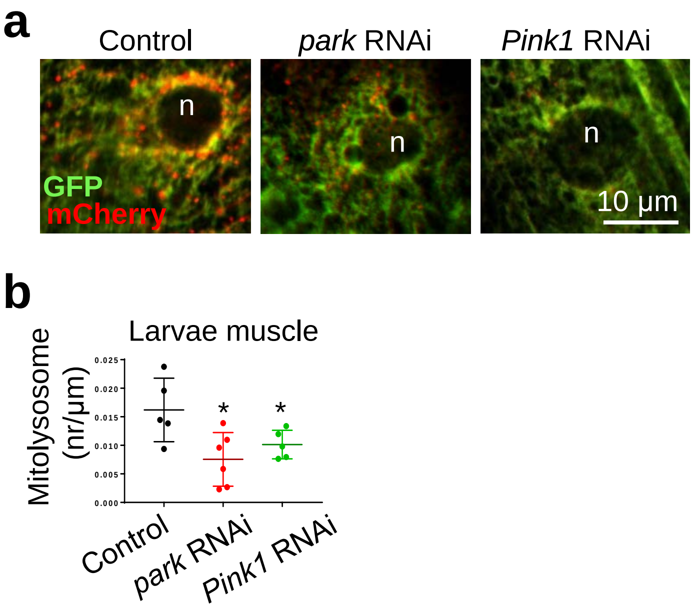

Supplemental Fig. 4

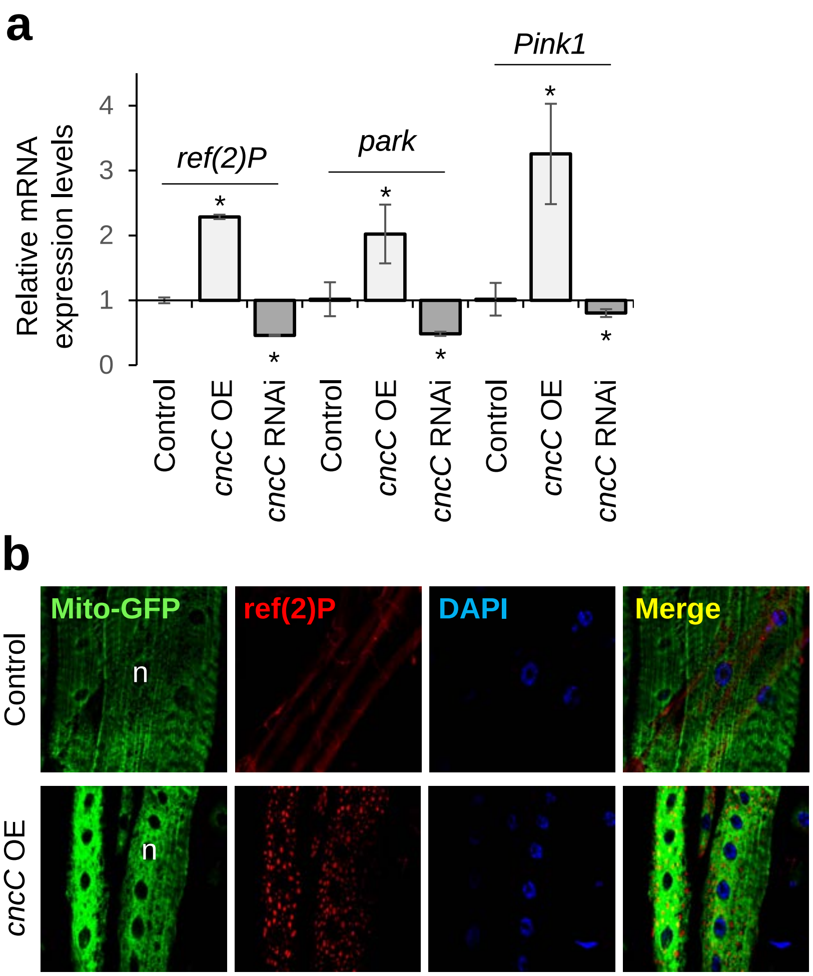

Supplemental Fig. 5

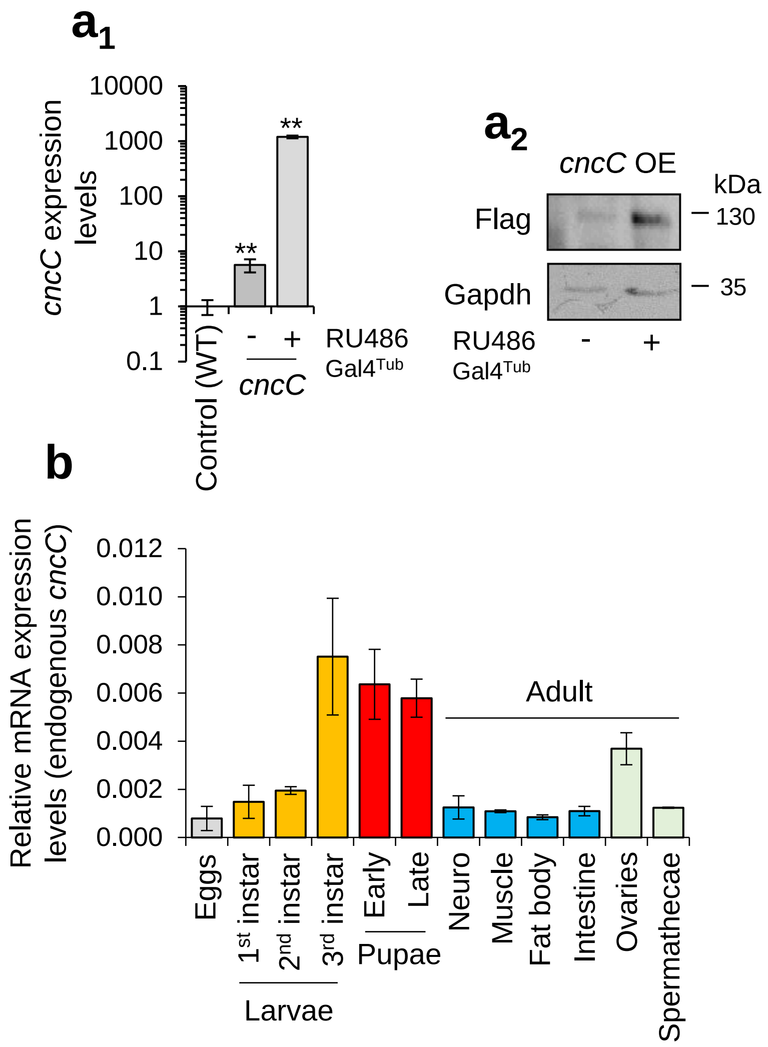

Supplemental Fig. 6

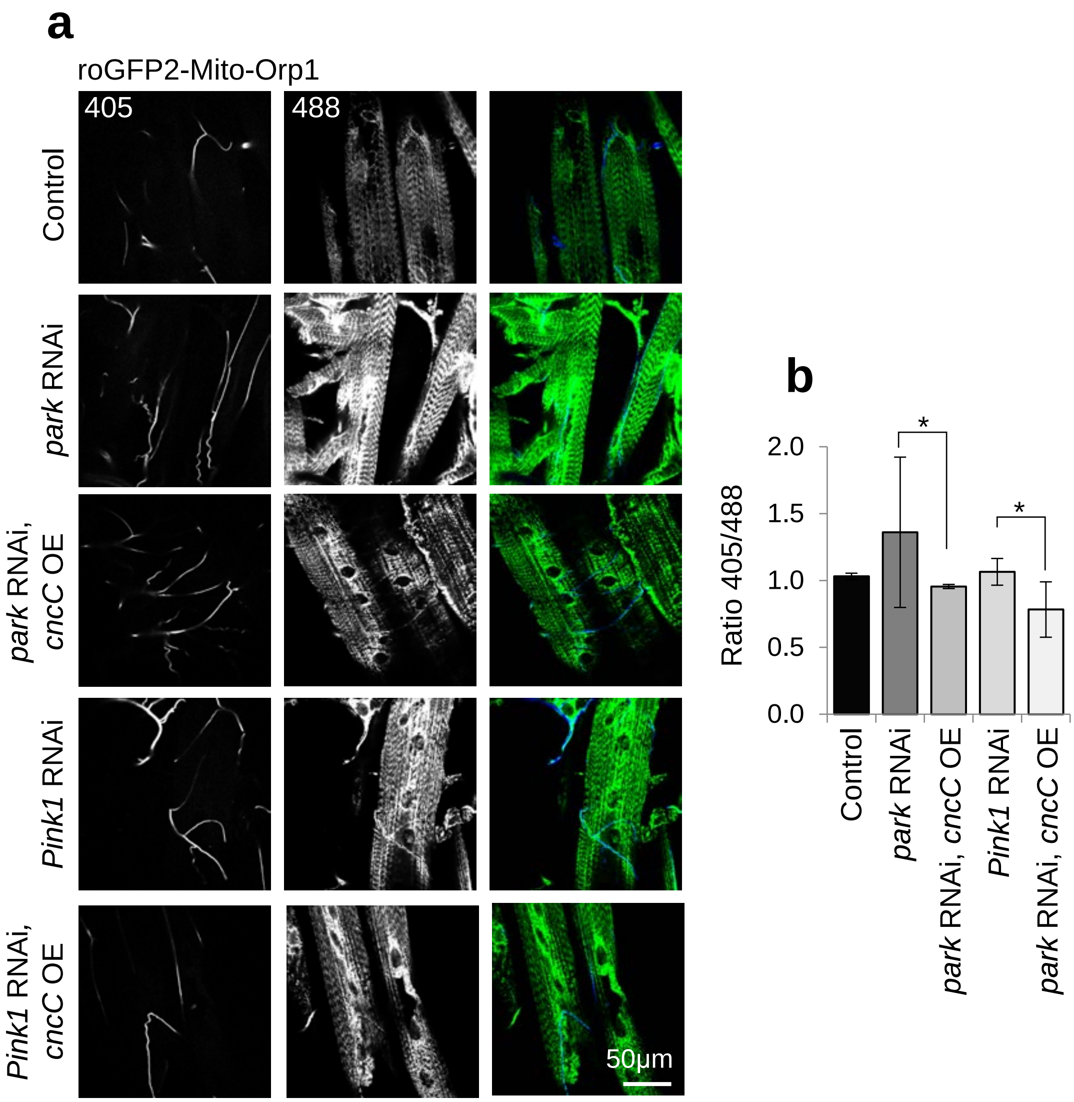

Supplemental Fig. 7

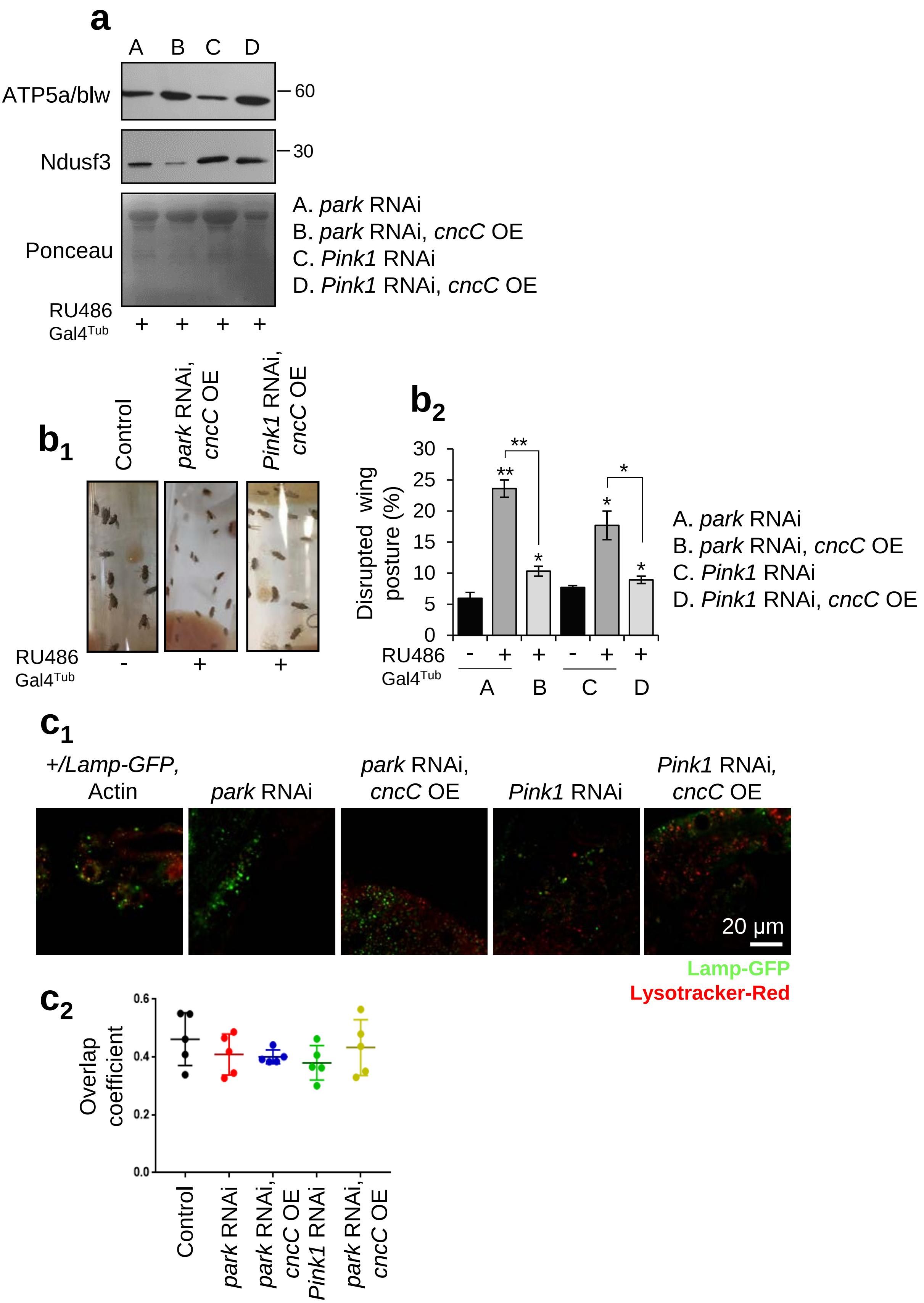

Supplemental Fig. 8

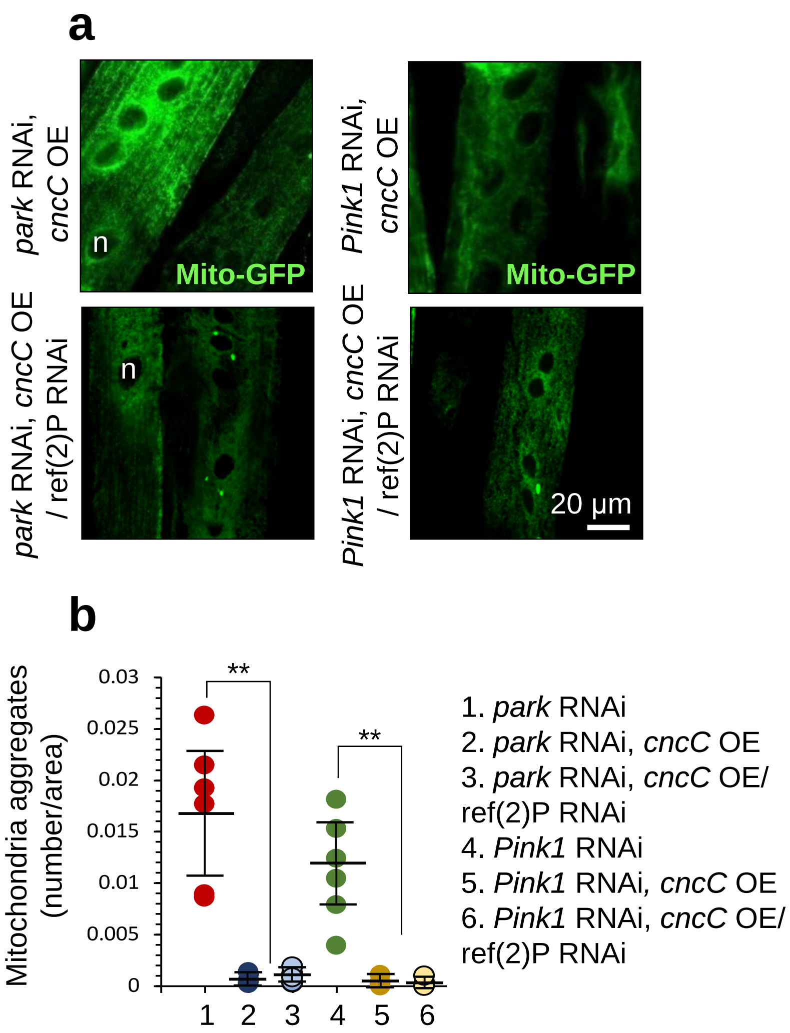

Supplemental Fig. 9

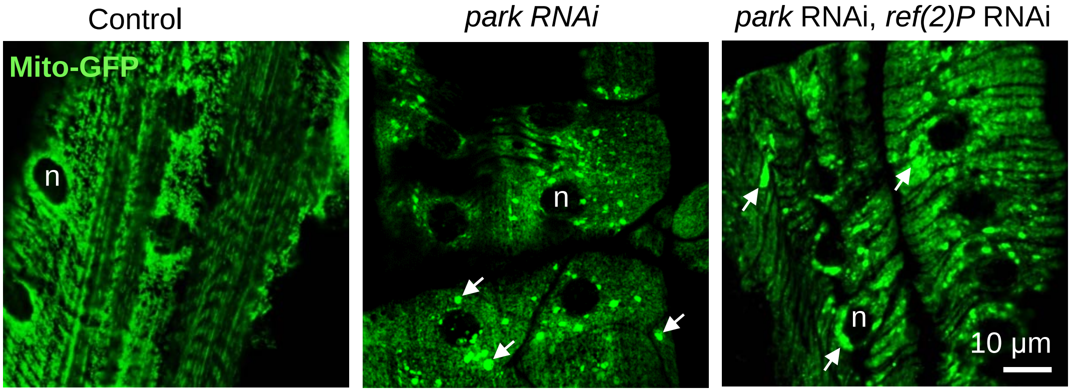

Supplemental Fig. 10

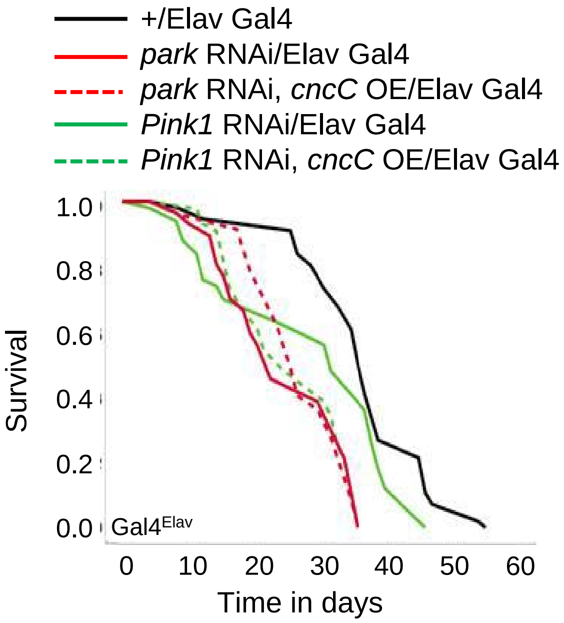

Supplemental Fig. 11

Supplementary Table S1. Summary of lifespan experiments.

Figure	Sample	Mean Lifespan (L.F) +/- s.e.m. (Days)		Median L.F +/- s.e.m. (Days)		% Median LF *vs*. control	Max (Days)	Log Rank P Value				
								park RNAi RU486 (-)	*park* RNAi RU486 (+)	*Pink1* RNAi RU486 (-)	*Pink1* RNAi RU486 (+)	*Total Animals Died/Total*
Figure 2E	*park* RNAi RU486 (-)	60	2.25	62	1.56	100	86		0.000			74/74
	park RNAi RU486 (+)	19	1.57	14	0.92	23	60	0.000				85/85
	Pink1 RNAi RU486 (-)	61	2.94	69	2.39	100	45				0.01	57/57
	Pink1 RNAi RU486 (+)	48	2.20	63	2.23	91	45			0.01		65/65

Figure	Sample	Mean Lifespan (L.F) +/- s.e.m. (Days)		Median L.F +/- s.e.m. (Days)		% Median LF *vs*. control	Max (Days)	+/Elav Gal4	*park* RNAi/Elav Gal4	*Pink1* RNAi/Elav Gal4	*park* RNAi, *cncC* OE/Elav Gal4	*Pink1* RNAi, *cncC* OE/Elav Gal4	*Total Animals Died/Total*
Figures S3, S11	+/Elav Gal4	35	1.29	35	0.68	100	54		0.000	0.035	0.000	0.000	56/56
	park RNAi/Elav Gal4	24	1.17	22	3.38	63	35	0.000			0.166		57/57
	Pink1 RNAi/Elav Gal4	28	1.81	31	2.12	80	45	0.035				0.000	50/50
	park RNAi, *cncC* OE/Elav Gal4	26	1.00	25	0.98	74	35	0.000	0.166				50/50
	Pink1 RNAi, *cncC* OE/Elav Gal4	25	1.23	23	2.90	71	35	0.000		0.000			50/50
